# Dr. Benjamin Lord

**Published:** 1902-06

**Authors:** 


### DR. BENJAMIN LORD.

Dr. Benjamin Lord died at his home, New York City, May 3, 1902.

Among the many who have passed from the activities of life few, it is surmised, will be more gratefully remembered than Dr. Benjamin Lord. His life had not been passed in the rush and turmoil common to ambitions men, but rather in the silent pathways where he sought to reach the truest place, that of a humble seeker after the highest good in his profession and in all the walks of life.

The International Dental Journal had no warmer friend than Dr. Lord. His personal efforts on its behalf were continuous to the last hours of his active life. The ethical principles it sought to inculcate were his principles, and his earnest hope was that these might take root in the dental mind as a moral power, leading it away from the influences of trade to a higher conception of those altruistic motives upon which all professions should be based.

To the writer he has been a friend and counsellor for many years, and to him, more than any other man, he owes much of that inspiriting force that always surrounds with a halo the good and true wherever found.

Dr. Lord did much to advance his profession. He rarely entered the arena of debate, but when he did it was always to aid to more practical excellence, as he understood the application of that word, to dental effort. While his methods may have been largely of the past, he was faithful to the present, and yearned with his true soul for a higher vantage-ground for his profession. He could not abide the selfishness so common among men, but with a broad liberality sought to lead to a better comprehension of that professional spirit that can alone unite all upon a foundation of success. The example of such men is the true touchstone, and those brought in contact with it are made to feel that its quiet influence has developed richer fruitage in the garden of their own spiritual natures. Others will garner the work of this man, but whatever may be said of his professional labors, to the writer his life will be a memory crowded with perpetual benedictions, in which the true and the beautiful will cluster, as the days and the years pass on into a world sadly forgetful of those who have helped to make life

> ". . . A suburb of the life elysian.
> Whose portal we call Death."

